# Automatic Speech Recognition in Noise for Parkinson's Disease: A Pilot Study

**DOI:** 10.3389/frai.2021.809321

**Published:** 2021-12-22

**Authors:** Alireza Goudarzi, Gemma Moya-Galé

**Affiliations:** ^1^Factorize, Tokyo, Japan; ^2^Department of Communication Sciences and Disorders, Long Island University, Brooklyn, NY, United States

**Keywords:** automatic speech recognition, multi-talker babble noise, Parkinson's disease, intelligibility, dysarthria

## Abstract

The sophistication of artificial intelligence (AI) technologies has significantly advanced in the past decade. However, the observed unpredictability and variability of AI behavior in noisy signals is still underexplored and represents a challenge when trying to generalize AI behavior to real-life environments, especially for people with a speech disorder, who already experience reduced speech intelligibility. In the context of developing assistive technology for people with Parkinson's disease using automatic speech recognition (ASR), this pilot study reports on the performance of Google Cloud speech-to-text technology with dysarthric and healthy speech in the presence of multi-talker babble noise at different intensity levels. Despite sensitivities and shortcomings, it is possible to control the performance of these systems with current tools in order to measure speech intelligibility in real-life conditions.

## Introduction

Parkinson's disease is the second most common neurodegenerative disorder, following Alzheimer's disease (Dorsey et al., 2007), with a prevalence of more than six million people worldwide (Dorsey et al., 2018). In the United States, the disease affects approximately one million individuals (its prevalence in 2020 was estimated to be 930,000), with numbers projected to increase to 1.2 million by 2030 (Marras et al., 2018). Although the average age of disease onset is 60 years (Ishihara et al., 2007), younger individuals (those in their 20s and 30s) may also be affected (Kostic, 2009).

One of the hallmarks of PD is the presence of dysarthria, a motor speech disorder, characterized by a significant reduction in vocal loudness (i.e., hypophonia), monopitch, hoarse and breathy vocal quality, misarticulations of consonants and vowels, short rushes of speech, and variable rate (Duffy, 2020). These deviant features of healthy speech have a significant impact on speech intelligibility, which refers to how an acoustic signal is decoded by a listener (Kent et al., 1989). Speech intelligibility is fundamental for success in communicative interactions (Kent and Kim, 2011) and, therefore, paramount for quality of life (Weismer, 2007). It is well known that ~90% of individuals with PD are likely to develop voice and speech problems during the course of the disease (Logemann et al., 1978) and that more than half of these speakers experience problems with intelligibility (Miller et al., 2007).

Speech perception is differentially affected when the acoustic signal occurs in noise (vs. in a quiet setting; Mattys et al., 2005), given the masking effects of noise on different segmental and suprasegmental cues in the speech signal. Intelligibility in individuals with PD is particularly affected in noisy environments, such as dining out at a restaurant or in social gatherings. What is more, a recent study showed that even those individuals with a mild speech disorder may experience a reduction in their intelligibility in the presence of background noise (Chiu and Neel, 2020). Multi-talker babble noise is a form of structured background noise that may mask a target speaker's voice. This noise is created by combining speech signals from multiple speakers. When multi-talker babble contains fewer speakers, noise is more likely to interfere with the foreground speaker, and, thus, there may be an increased difficulty to decode the target speaker's exact utterance.

In the context of a chronic illness, such as PD, collaborative disease management encourages individuals with PD to closely work with their treating clinicians to maintain and/or improve their well-being (Lyons, 2004). One of the tenets of this approach is the notion of self-management, which corresponds to the patient's ability to observe a given behavior and react or problem-solve according to such observation (Lorig, 1993). Dysarthria latency in PD averages 7 years post disease onset (Müller et al., 2001). Therefore, when considering our patients within a collaborative management approach, self-management techniques can serve to establish preventative measures for speech and intelligibility degradation and/or control measures of intelligibility levels if speech deficits already exist. As shown in Hayes (2002) survey of 120 individuals with PD on a variety of self-management characteristics, knowing how to respond to worsening of disease symptoms and when to seek medical advice are crucial aspects in patients' well-being.

### Clinical Applications of Artificial Intelligence

The use of artificial intelligence (AI) for automatic speech recognition (ASR) has greatly evolved in the past years. This technological advancement can be experienced in our daily lives, from captions in movies, digital assistants (e.g., Siri) in mobile phones to home appliances (e.g., Alexa). The use of AI has facilitated communication for a wide range of individuals, including those with hearing loss and speakers with motor impairments, hence its benefits for improved quality of life seem, at the very least, promising. For those individuals with speech disturbances, such as those caused by laryngectomy (Schuster et al., 2006), head and neck cancer (Maier et al., 2010), cleft palate (Maier et al., 2006) or oral cancer (Maier et al., 2007), ASR has also been shown to be effective in estimating speakers' intelligibility deficits (Tu et al., 2016). For individuals with dysarthria, however, ASR research has been more limited (Christensen et al., 2012; Sharma and Hasegawa-Johnson, 2013) and it has highlighted the high degree of variability inherent in dysarthric speech (Tu et al., 2016).

Despite the undeniable success of deep neural networks (DNN) in enhancing the quality of ASR (Amodei et al., 2016; Arik et al., 2017), these systems remain sensitive to noise in input signals. Typical training of speech recognition systems uses samples recorded in a quiet environment. If noise is implemented, however, it is either not “natural” (Zhang et al., 2017), or only occurs during the training phase (Chan et al., 2016). Therefore, the effect of unstructured and structured noise in real-life speech recognition remains largely unknown. Additionally, it has been noted that DNNs may behave unpredictably when provided with perturbed or out-of-distribution samples (Cisse et al., 2017; Eykholt et al., 2018). Research to improve the robustness of ASR in noisy environments is an active research area (Richey et al., 2018; Mošner et al., 2019). Therefore, understanding the sensitivity of DNNs to various application-specific types of noise and establishing protocols to ameliorate response variability can help generalize AI to real-life applications.

The goal for this pilot study was to measure speech intelligibility in individuals with Parkinson's Disease using ASR in noise. To this end we report the sensitivity of Google Cloud speech-to-text API, a prominent provider of ASR, to a specific type of background noise, multi-talker babble, which is commonly implemented in the study of dysarthria (Moya-Galé et al., 2018; Chiu and Neel, 2020).

## Materials and Methods

This study was approved by the Institutional Review Board at Long Island University, Brooklyn, NY.

To perform this pilot study, we developed a web application intended to be used on participants' cell phones. The web application prompted each user to record their voice while reading a predetermined set of sentences. The sentences were sent to a backend server stored for post processing. The recordings were then resampled, mixed with multi-talker babble noise at a given signal-to-noise ratio (SNR) and sent to Google speech-to-text API for the recognition phase. The recognition results were then used to calculate word-error-rate against the original sentences. Each of these sections is detailed below.

### Participants

Five individuals with PD (3 females, 2 males; mean age = 71.2 years, SD = 13.07 years, age range = 49–81 years) participated in this study. Inclusion criteria for participation included: (1) having a medical diagnosis of PD, (2) having a stable antiparkinsonian medication, (3) being a native English speaker, and (4) having experienced changes in voice and/or speech or reporting voice or speech as a current concern. Participants who had undergone deep brain stimulation surgery or received individual, intensive voice treatment within the last 2 years were excluded. Five neurologically healthy adults (3 females, 2 males; mean age = 63.2 years, SD = 13.14 years, age range = 40–71 years) served as experimental controls. Background information on individuals with PD and healthy controls is provided in Table 1.

**Table 1:** Participant information, including age, sex, years since PD diagnosis and dysarthria severity.

**Participant**	**Group**	**Age**	**Sex**	**Years since PD diagnosis**	**Dysarthria severity**
P1	PD	81	M	8	Moderate
P2	PD	71	F	4	Mild
P3	PD	76	F	9	Mild
P4	PD	48	M	7	Mild
P5	PD	79	M	10	Moderate
P6	HC	66	M		
P7	HC	71	F		
P8	HC	71	M		
P9	HC	68	F		
P10	HC	40	M		

*PD, Parkinson's disease; HC, healthy control; M, male; F, female*.

### Procedure

Recordings were self-paced, and they were completed in the participants' homes, in a quiet space. The evaluator (second author) met with participants over Zoom to instruct them on the recording procedures and clarify any questions. Careful instructions were provided so that speakers maintained a constant distance of 8 cm (~3.15 inches) between their mouths and the recording device. Carepartners were recruited to assist speakers when PD-related difficulties hindered proper recording procedures (e.g., tremors).

### Speech Stimuli

A data set of 50 sentences was created for this pilot study. Sentences were grammatically and semantically correct (e.g., *Make the most of your time*; *The schedule is flexible, but the salary is low*), varied in length, from 5 words to 9 words, and contained high frequency English content words from the English Lexicon Project (Balota et al., 2007). Speakers accessed our customized web-based app, *Understand Me for Life*, from their phones and were provided with a unique user code. A brief familiarization task consisting of three sentences was subsequently completed in order to ensure participants' full comprehension of how to utilize the recording interface. They were instructed to read each sentence using their typical or habitual voice. Following the familiarization phase, the app provided a list of five randomized sentences to read. The task took ~15 mins to complete.

### Sample Rate Adjustment

ASR performance was shown to be very sensitive to sample rate. Due to storage space considerations and adhering to prior studies we had initially converted all the recordings to 8 kHz single channel audio. Although to the human ear there is very little difference between this format and the original 48 kHz recording, ASR is notoriously unforgiving. Although Google ASR API provides an enhanced speech recognition model for telephone audio, this API did not improve the performance for our recordings, as detailed in the Results section. We therefore ensured both the noise and audio recordings were stored in a 48 kHz single channel format.

### Multi-Talker Babble Noise

Multi-talker babble noise was created to emulate the cocktail party effect (O'Sullivan et al., 2015), where certain vowels and consonants blend with the background speech from nearby speakers. This type of noise was generated by recording a 30-s sample from NPR when a single speaker was speaking. The audio was hand selected to avoid recording any overpronunciation, exaggeration or sudden changes in vocal intensity (e.g., driven by the context of program). Prolonged silences (i.e., over 500 ms) were trimmed and equalization of the audio spectrum in a moving window was subsequently performed. The equalized audios were combined to create the final audio file, which contained an equal number of male and female speakers (Moya-Galé et al., 2018), which resulted in 10-talker babble (5 males, 5 females; Chiu and Neel, 2020).

### The Use of Phrase Hints

Despite impressive performance of speech recognition in laboratory environments, there are characteristic differences in how these systems work compared to human auditory perception that severely affect ASR performance in noisy backgrounds. For example, the reliance on context and attention in human hearing (O'Sullivan et al., 2015) helps a human listener subconsciously guess a partially inaudible word or clearly distinguish a speaker's voice from the background noise even in the presence of highly structured noise, such as multi-talker babble. Google ASR API allows submission of a list of words to be detected in the sample audio. Not only does this better align ASR with human listener performance, but it also helps avoid many of the common ASR challenges, such as mismatched verb tense (e.g., develop for developed), plural vs. singular words (e.g., car for cars) or homonyms (e.g., sea levels vs. C-levels).

### Accuracy Calculation

For a given utterance *S* and the corresponding ground sentence *T* we first pad the shorter of the two with space until both *S* and *T* were of equal length *L*. We codified each word in *S* with *w*_*s*_and each word in *T* with *w*_*t*_where *s* and *t* were numbers from 0 to *L*−1. We then calculated the accuracy as the percentage of matching words between *S* and *T* in a suitable alignment as follows:


$$\begin{array}{l} f\left(S,T\right)=argmax_{s,t}\frac{100}{L}\overset{L-1}{\underset{s=0}{\sum }}\overset{L-1}{\underset{t=0}{\sum }}\sigma \left(w_{s},w_{t}\right) \end{array}$$

where σ(*w*_*s*_*,w*_*t*_) = 1 if *w*_*s*_ = *w*_*t*_ and 0 otherwise. This setup avoids rendering a score to words that appear in both *S* and *T* but out of order.

## Results

### The Effect of Downsampling Without Noise

Sampling rates of 8 and 48 kHz were contrasted to assess ASR performance in a quiet environment. A Kruskal-Wallis test was conducted to examine downsampling effects on ASR accuracy scores. Downsampling at 8 kHz yielded significantly worse performance than ASR at 48 kHz [$\chi_{\left(1\right)}^{2}$ = 9.153, *p* = 0.002]. Thus, a sampling rate of 48 kHz was implemented in the subsequent experiments.

Table 2 provides intelligibility accuracy results from sampling rates at 8 and 48 kHz.

**Table 2:** Automatic speech recognition accuracy scores at a sampling rate of 8 kHz and a sampling rate at 48 kHz.

**Sampling rate at 8 kHz**			
	**Speakers with PD**	**Healthy controls**	**Total**
Mean accuracy (%)	90	92	91
Standard deviation	14	13	13
**Sampling rate at 48 kHz**			
Mean accuracy (%)	96	100	98
Standard deviation	8	0	6

### The Effect of Multi-Talker Babble Noise

**Figure 1:**
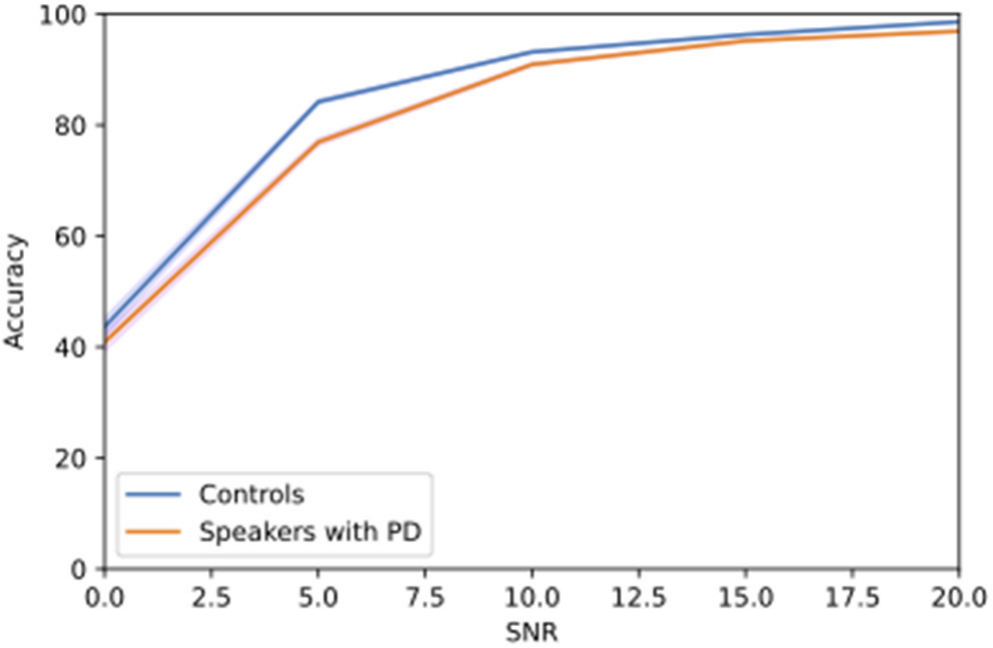
Change in accuracy of speech recognition with no hinting for speech embedded in 10-talker babble noise at different SNR levels.

Findings from different SNRs revealed that average ASR performance started declining at 10 dB SNR, with a more noticeable reduction in accuracy scores at 5 dB SNR (Figure 1).

Without hinting, no significant difference in ASR accuracy scores was found between speakers with PD and healthy controls across different SNRs (*p* > 0.05).

### The Use of Phrase Hints

**Figure 2:**
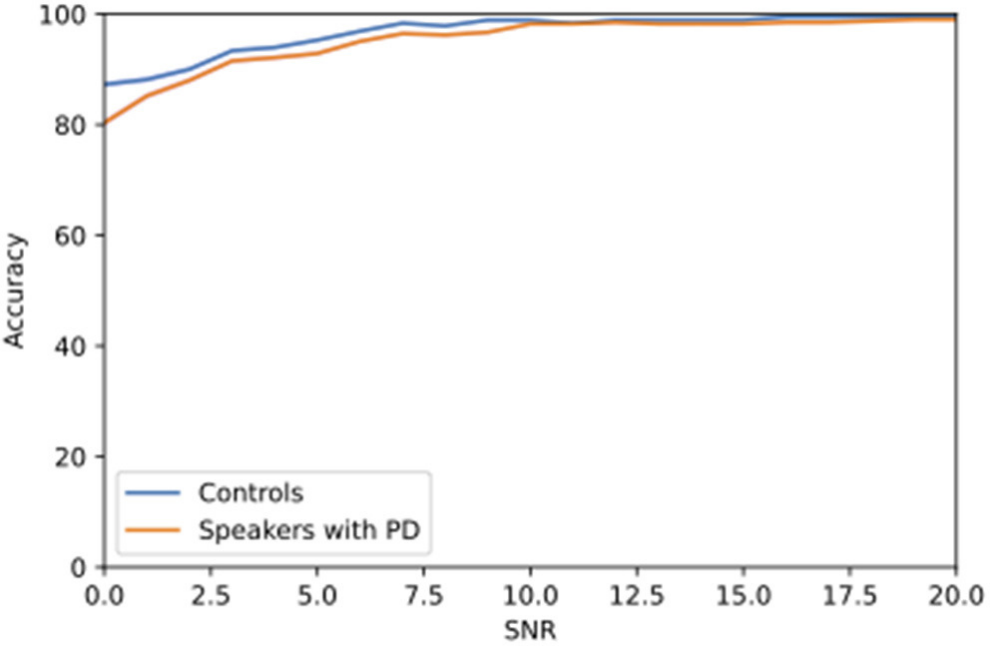
Change in accuracy of speech recognition with hinting for speech embedded in 10-talker babble noise at different SNR levels.

The use of hinting rendered an improvement in ASR in lower SNRs compared to the previous condition (Figure 2). A Kruskal-Wallis test yielded a significant difference in ASR performance between the hinting and no hinting conditions at 0 dB SNR [$\chi_{\left(1\right)}^{2}$ = 29.225, *p* < 0.001].

Additionally, a significant difference in ASR accuracy scores in 10-talker babble noise was found between speakers with PD and healthy controls at 0 dB SNR [$\chi_{\left(1\right)}^{2}$ = 5.278, *p* = 0.022]. No significant difference was found between the two groups at the other SNRs (*p* > 0.05).

## Discussion

This pilot study examined the voice recognition accuracy of a popular speech-to-text service provided by Google Cloud Platform in two groups of speakers, individuals with PD and healthy controls. Our goal was to determine the feasibility of implementing this service in the development of assistive technologies for people with PD, whose voice and speech difficulties may significantly decrease their intelligibility in noisy settings. To that end, ASR aimed at replicating real-life challenges, such as the presence of background multi-talker noise embedded within the speaker's speech signal.

Our initial experiment investigated the effects of downsampling in a quiet condition, as this has been reported as a potential factor affecting ASR. As has been advised by Google, the downsampling under 16 kHz significantly reduced the accuracy of speech recognition. The use of multitalker babble noise was subsequently implemented to determine ASR accuracy for speakers with PD and healthy controls in different levels of background noise. As expected, results revealed differential AI performance depending on the SNR level, with higher noise levels corresponding to a substantial decrease in ASR accuracy (~40%) in both groups. Additionally, without phrase hinting, no difference could be detected between ASR accuracy scores for speakers with PD and healthy controls. A subsequent application of hint phrases to facilitate ASR and emulate human listeners' performance yielded a statistically significant improvement in ASR accuracy scores at the most challenging noise condition, 0 dB SNR. Furthermore, under this condition the algorithm was also able to differentiate between individuals with PD and healthy controls.

This work expands traditional research on intelligibility in dysarthric speech, which traditionally relies on human transcriptions of phrases or sentences presented in noise, to incorporate AI. In particular, this pilot study showed that given the current tools, it is possible to control the behavior of ASR to approximate that of human listeners in its sensitivity to noisy backgrounds. This opens the door to further studies in this area and development of assistive technologies using existing AI technologies. The pilot of our current web-based app, *Understand Me for Life*, therefore, shows promise in the ability of the program to simulate real-life intelligibility challenges posed by ambient noise in the process of speech recognition and in providing individuals with PD with a self-monitoring and easy to use tool to track their intelligibility changes over time.

## Data Availability Statement

The raw data supporting the conclusions of this article will be made available by the authors, without undue reservation.

## Ethics Statement

The studies involving human participants were reviewed and approved by Institutional Review Board at Long Island University-Brooklyn. The patients/participants provided their written informed consent to participate in this study.

## Author Contributions

GM-G and AG conceptualized the study and co-led the write-up of the manuscript. AG developed the technology for the app and computed results. GM-G developed the data set, recruited participants for the study, and trained them to conduct the voice recordings on their phones. Both AG and GM-G contributed to the final version of the manuscript.

## Funding

This study was funded by a research grant from the Michael J. Fox Foundation, awarded to GM-G.

## Conflict of Interest

AG was employed by company Factorize. The remaining author declares that the research was conducted in the absence of any commercial or financial relationships that could be construed as a potential conflict of interest.

## Publisher's Note

All claims expressed in this article are solely those of the authors and do not necessarily represent those of their affiliated organizations, or those of the publisher, the editors and the reviewers. Any product that may be evaluated in this article, or claim that may be made by its manufacturer, is not guaranteed or endorsed by the publisher.
